# *L*-Theanine Prevents Autophagy Impairment to Counteract Cadmium-Induced Hepatic Mitochondrial Dysfunction, Ferroptosis, and Glucolipid Dysmetabolism in Mice

**DOI:** 10.3390/nu18183098

**Published:** 2026-09-21

**Authors:** Qiuyan Ban, Wenjing Chi, Qiong Wang, Junsheng Li, Yao Xia, Yue Meng, Mengru Li, Renliang Zhao, Yiding Yu, Zhipeng Kan, Ning Li, Yan Ma, Xianqing Huang, Dongxu Wang, Guangshan Zhao

**Affiliations:** 1 Department of Tea Science, College of Horticulture, Henan Agricultural University, Zhengzhou 450046, China; 2 Key Laboratory of Food Nutrition and Functional Food of Hainan Province, School of Food Science and Engineering, Hainan University, Haikou 570228, China; 3 State Key Laboratory of Tea Plant Germplasm Innovation and Resource Utilization, Tea Research Institute, Chinese Academy of Agricultural Sciences, Hangzhou 310008, China; 4 Henan Engineering Technology Research Center of Food Processing and Circulation Safety Control, College of Food Science & Technology, Henan Agricultural University, Zhengzhou 450002, China; 5 School of Grain Science and Technology, Jiangsu University of Science and Technology, Zhenjiang 212100, China

**Keywords:** *L*-theanine, cadmium, autophagy, mitochondrial dysfunction, ferroptosis, glucolipid dysmetabolism

## Abstract

Background: Cadmium (Cd) exposure impairs the autophagic response, a core mechanism for cell preservation and organismal homeostasis, eventually resulting in metabolic disturbances, mitochondrial dysfunction, and oxidative stress. L-theanine (LT) exhibits multiple health benefits with a high safety profile. However, the effects of LT on Cd exposure-induced impairments in autophagy and autophagy-related physiological functions remain unclear. Methods: This study investigated the effects of LT on the survival time of mice acutely exposed to Cd and the regulating effect of LT on autophagy and subsequent metabolic dysfunction in mice subjected to subchronic Cd exposure. Results: The results showed that acute Cd exposure resulted in 100% mortality within 8 h. However, LT treatment significantly prolonged the median survival time of mice from 4 h to 16 h and reduced the mortality to 60%. In the context of subchronic Cd exposure, autophagy was inhibited, as evidenced by the downregulation of the AMPK/mTOR signaling pathway mediated by DPP-4 and SIRT1. This exposure also promoted lipid peroxidation and ferroptosis, indicated by the inactivation of the AMPK/*p*-ACC axis and marked alterations in ferroptosis markers. Furthermore, mitochondrial dysfunction was suggested by the downregulation of the SIRT1/PGC-1*α*/Nrfs/TFAM signaling pathway and reductions in COX and SDH activities. Additionally, glucolipid metabolism was impaired, as indicated by the downregulation of the AMPK and PI3K/AKT signaling pathways and elevated lipid and glycogen accumulation in the liver of mice. LT significantly decreased the level of DPP-4 and upregulated SIRT1, leading to the activation of AMPK. This activation restored autophagy response by downregulating mTOR, promoted energy homeostasis by reducing lipid biosynthesis and enhancing fatty acid oxidation, blocked lipid peroxidation and ferroptosis through the phosphorylation of ACC at the serine 79 site and reducing the ubiquitination-mediated degradation of GPX4, and maintained mitochondrial function by upregulating the PGC-1*α*/Nrfs/TFAM signaling pathway. Conclusions: Collectively, LT effectively ameliorates mitochondrial dysfunction, ferroptosis and glucolipid dysmetabolism in Cd-exposed mice, and these effects are accompanied by findings consistent with modulation of autophagy-related signaling in the liver.

## 1. Introduction

Nowadays, 14 to 17% of cropland worldwide is affected by at least one toxic metal, including cadmium (Cd), arsenic, cobalt, chromium, copper, nickel, and lead, causing 0.9 to 1.4 billion people to live in regions of heightened public health and ecological risks [1]. Globally, Cd exceedance for agricultural soil is the highest of these toxic metals, reaching 9.0% [1]. Toxic metal pollution not only has considerable impacts on food production and food safety but also ultimately affects human health due to bioaccumulation in the food chain. Given its remarkably long biological half-life in humans (20–40 years) [2], Cd is easily absorbed and accumulated in the body and triggers deleterious health impacts, even at low-dose exposure, and it has been demonstrated to be associated with the occurrence of liver disease, cardiovascular diseases, diabetes mellitus, osteoporosis, neurodegeneration and reproductive issues. High Cd exposure markedly increases mortality by approximately 17% [3]. Of these at-risk populations, dietary intake accounts for ~90% of total Cd exposure (global average ~2.20 μg/kg bw/day) [4], while smoking one pack daily adds 2–4 μg [5]. Occupational exposure, though affecting fewer people, involves much higher levels—about 207,350 workers in the EU and 525,000 in the US—historically contributing to an estimated 2000 deaths and 47,000 disability-adjusted life years globally in 2015. Thus, both environmental (mainly foodborne) and occupational sources drive the substantial global Cd burden.

The adverse health effects of Cd exposure are mainly attributed to its metal toxicity, namely mitochondrial dysfunction, endocrine disruption, endoplasmic reticulum stress, oxidative stress and inflammation, and the regulating effects on gut microbiota and intestinal physiology [3]. Autophagy, a dynamic recycling system that produces new building blocks and energy for cellular renovation, is a core molecular pathway for the preservation of cellular and organismal homeostasis [6,7]. It has a dual role where it acts as a survival mechanism when autophagy can reduce damage and in programmed cell death when the overall dose and time exceed safety thresholds [8]. Autophagy dysfunction is one of the main mechanisms underlying Cd-induced cytotoxicity [9]. Impairment of autophagy responses and mutations in autophagy-related processes promote or aggravate disease, such as metabolic, cardiovascular, neurodegenerative, musculoskeletal, renal and pulmonary disorders [6,7]. Cd is one of the potent inducers of autophagy, which has been suggested to be an adaptive stress response to allow metal-exposed cells to survive in hostile environments [10]. Numerous reports have shown that Cd exposure promotes autophagy through classical molecular pathways involving the reactive oxygen species (ROS)-dependent signaling pathways, the endoplasmic reticulum stress pathway, the mammalian target of rapamycin (mTOR) pathway, and the Beclin-1 family [9]. However, several reports have shown that Cd exposure suppresses autophagy by downregulating the sirtuin 1 (SIRT1)/5′ AMP-activated protein kinase (AMPK) signaling pathway, the upstream of mTOR, causing oxidative stress, mitochondrial dysfunction and metabolic disturbances [11,12,13,14]. An appropriate autophagic response is of critical importance for the later stages of autophagy [12,14].

Dimercaptosuccinic acid, dimercaptopropane sulfonate and diethyl dithiocarbonate are the most effectively chelators for detoxification of acute Cd exposure, and they have been shown to be therapeutically beneficial in humans and animals when used according to the protocols specified by physicians or veterinarians [15,16,17]. However, these compounds often show a high toxicity profile and cause a significant loss of certain essential trace elements, such as K^+^, Ca^2+^, and Mg^+^ [18]. Moreover, so far, there are no highly effective approaches for the intervention of subchronic and chronic Cd toxicity. Therefore, the development of novel strategies for mitigating or eliminating Cd toxicity is required.

Natural compounds with the ability to restore autophagy flux and mitigate Cd toxicity represent a promising intervention strategy [12]. Tea, made from the plant *Camellia sinensis* L., is a health-promoting beverage rich in diverse bioactive compounds and is consumed worldwide [19,20]. *L*-theanine (LT), a non-protein, water-soluble amino acid, is a unique amino acid in the tea plant (*Camellia sinensis* L.) with multiple beneficial functions, including metabolic regulatory, liver-protective, anti-obesity, antioxidant, and neuro-protective effects [21], as well as autophagy-modulating effects. Namely, LT ameliorates substance P-induced bladder hyperactivity through the inhibition of microtubule-associated protein 1 light chain 3-II (LC3-II)-mediated autophagy in rats [22]. LT promotes proliferation, together with accelerated neurogenesis in murine undifferentiated neural progenitor cells, by activating the mTOR signaling pathway, which participates in cell growth and autophagy [23]. Adipose stem cells preincubated with LT exert liver regeneration via suppression of autophagy in rats with *N*-nitrosodiethylamine-induced liver damage [24]. LT reduces the oxidative stress of the small intestine and maintains the normal morphology and function of mitochondria by enhancing Bcl-2 expression and inhibiting autophagy of the mitochondrial pathway in mice [25]. It also protects against neurotoxicity through inhibiting oxidative damage and tau hyperphosphorylation in mice [26] and PC12 cells apoptosis by downregulating the mitochondria-mediated pathway induced by Cd [27]. Furthermore, LT shows a high safety profile. No adverse reaction was observed post oral administration of 4000 mg/kg bw/day for 13 weeks when LT was administered as a dietary admixture in male and female rats [28]. The US Food and Drug Administration certified LT as a generally recognized safe substance, and the recommended daily intake for humans is 1200 mg [29]. These reports suggest that LT has the potential to modulate or eliminate the harmful effects, including autophagy impairment, metabolic disturbances, and oxidative damage, caused by Cd exposure.

The liver serves as a key target organ for Cd toxicity. Protecting the liver from Cd toxicity [30,31] is vital for maintaining its normal metabolic and detoxification functions. Herein, we tested the hypothesis that LT can maintaining hepatic autophagy response and counteract the detrimental effects induced by Cd exposure in mice. Specifically, this study investigated the impact of Cd on the autophagic response and examined the regulatory effects and underlying mechanisms of LT in this process. We focused on elucidating how LT alleviates Cd-induced suppression of autophagy, and the consequent mitochondrial dysfunction, ferroptosis, and glucolipid dysmetabolism.

## 2. Materials and Methods

### 2.1. Materials

CdCl_2_ and LT were purchased from Macklin (Cat C805625) (Shanghai, China) and Aladdin (Cat 3081-61-6) (Shanghai, China), respectively. Other materials were of the highest grade available from Chinese market.

### 2.2. Animals

Male and 3-week-old Kunming mice with an average body weight of 14 ± 2 g were purchased from Laboratory Animal Center, Zhengzhou University. The feeding temperature was 23 ± 2 °C, relative humidity was 50 ± 5%, and light time was 12 h/d, while free diet (normal diet, total energy is 3530 kcal/kg, with 67.4%, 20.6%, or 12.0% of energy derived from carbohydrate, protein, or fat, respectively; Cat 1010088, Jiangsu Synergy Pharmaceutical Bioengineering Co., Ltd., Shanghai, China; Supplementary Table S1) and water were provided to the mice. This study was carried out in compliance with the ARRIVE guidelines, and the research protocols and experiments were approved by the Laboratory Animal Ethics Review Committee of the Key Laboratory of Animal Immunology, Henan Academy of Agricultural Sciences (approval number LLSC410240005, 6 January 2024). All experimental manipulations were performed in accordance with the 3R guidelines (replacement, reduction, and refinement), striving to minimize animal usage and reduce distress. Mice were excluded if they (1) exhibited signs of illness or a body weight loss exceeding 20% during the one-week acclimation period; (2) were euthanized prematurely during Cd exposure due to humane endpoints, such as severe weight loss, impaired mobility, or moribund condition; or (3) died unexpectedly before the scheduled sampling time point, preventing the collection of liver tissue and serum samples. No animals were excluded in this study.

### 2.3. Experimental Design

Before the experiment, the mice were acclimated to the facility for one week. This animal experiment aims to investigate whether LT protects against Cd-induced mortality in mice. Twenty-four mice were randomly divided into 3 groups (n = 8/group/cage): (1) the control group (Ctrl), (2) the CdCl_2_ treatment group (Cd) (8 mg/kg, *i.p.*), and (3) the LT + CdCl_2_ treatment group (LT) (200 mg/kg LT, *i.g.*). The LT treatment group was orally gavaged with LT (200 mg/kg) for 3 days consecutively before injection with 8 mg/kg CdCl_2_. The sample size (n = 8 per group) was determined based on the effect sizes observed in our preliminary experiments and the sample sizes commonly used in comparable studies of Cd-induced hepatotoxicity in mice. The total number of animals used was 32.

To investigate the mechanism of LT alleviating the metabolic disorders caused by Cd, we performed a separate subchronic experiment. Of note, the administration routes differed intentionally between the two experimental settings. In the acute mortality study, Cd was given via intraperitoneal injection to ensure complete and rapid systemic absorption, which is critical for accurate mortality endpoint assessment. In the following chronic study, oral gavage was adopted to mimic the natural route of human dietary exposure to Cd, and LT was supplied via drinking water to minimize stress associated with long-term daily manipulations. The Cd exposure levels used in this study—particularly after body surface area-based allometric conversion—are higher than the general population’s daily dietary exposure levels. However, these doses are designed to model high-risk populations (such as occupationally exposed workers or residents of contaminated areas) and to provide sufficient margins for clearly observing pathophysiological changes and the protective effects of LT in the animal model. Although the LT dose used in this study is higher than common human supplementation doses, extensive human clinical trials have demonstrated that LT is safe and well-tolerated at doses up to 900 mg/day [32]. This provides a solid safety foundation for its potential clinical translation. Moreover, the doses of Cd and LT used in this study were determined as previously reported by us [33,34].

For the chronic experiment, the mice were randomly divided into 4 groups (n = 9/group/cage): (1) the control group (Ctrl), (2) the CdCl_2_ treatment group (Cd) (2.5 mg/kg, *i.g.*), (3) the LT (1 mg/mL in drinking water) + CdCl_2_ treatment group (L-LT), and (4) the LT (2 mg/mL in drinking water) + CdCl_2_ treatment group (H-LT). All mice of the three treatment groups were gavaged every day for 10 weeks. Before gavage with CdCl_2_, the mice in LT treatment group drank water containing LT (1 or 2 mg/mL) for 2 days. All mice were sacrificed after receiving treatment continuously for 10 weeks, and the liver and serum were collected and frozen immediately in −80 °C for subsequent study. The doses used of Cd (8 mg/kg or 2.5 mg/kg CdCl_2_) in this study refer to CdCl_2_ but not to the content of elemental Cd. The sample size (n = 9 per group) was determined based on the effect sizes observed in our preliminary experiments and the sample sizes commonly used in comparable studies of Cd-induced hepatotoxicity in mice. The total number of animals used was 36.

To control for confounding, cage positions on the rack were cyclically shifted horizontally every 3 days to mitigate fixed-location biases (noise, light, ventilation). Gavage and weighing were consistently conducted between 10:00 and 12:00 a.m. daily, and the daily sequence of cage handling was re-randomized using a random number table to avoid operator-dependent variability. No blinding was applied at any stage of the experiment. The investigator who generated the randomization sequence, the personnel who performed animal treatments, the outcome assessors, and the data analyst were all aware of group allocation.

Primary outcome measure: This was a hypothesis-testing study. The primary outcome measure was prespecified as hepatic autophagy prior to data collection. No a priori sample size calculation was performed because no reliable effect size data were available for LT in Cd-induced hepatic injury; therefore, the sample size was not determined by the primary outcome measure. The sample size used in this study was based on our preliminary experiments and sample sizes commonly used in comparable studies of Cd-induced hepatotoxicity in mice. Secondary outcomes included autophagy-related proteins, ferroptosis markers, and glucolipid metabolism parameters.

### 2.4. Biochemical Assays

Commercial assay kits were used to measure the concentrations of free fatty acids (FFA, Cat BC0595, Solarbio), glycogen (Cat BC0345, Solarbio) (Beijing, China), triglyceride (TG, Cat A110-1-1), total cholesterol (TC, Cat A111-1-1), high-density lipoprotein cholesterol (HDL-C, Cat A112-1-1), malondialdehyde (MDA, Cat A003-1), alkaline phosphatase (AKP, Cat A059-2-2), catalase (CAT, Cat A007-1-1) activity, reduced glutathione (GSH, Cat A006-2-1) (Nanjing Jiancheng Bioengineering Institute) (Nanjing, China), cyclo-oxygen-ase (COX, Cat YJ34434), succinate dehydrogenase (SDH, Cat YJ34650), and dipeptidyl peptidase-4 (DPP-4, Cat YJ001952) (Yuanjubio, Shanghai, China) according to the manufacturers’ instructions. The contents of Fe^2+^ in serum and tissues were detected using a ferrous iron colorimetric assay kit (E-BC-K773-M, Elabscience, Wuhan, China) according to the manufacturer’s instruction.

After the liver tissues were fixed within 4% paraformaldehyde for 24 h, the samples underwent a conventional process with graded alcohol and xylene for tissue dehydration and clearing. Then, the samples embedded in paraffin were sectioned into 5 µm slices, stained with H&E, PAS, and Oil red O, and examined using a Motic VM1 digital slide scanner (Motic China Group Co., Ltd., Xiamen, China; model VM1) equipped with a Moticam Pro 285E camera (2/3” CCD, 1360 × 1024 pixels). Digital scanning and image acquisition were performed with the Motic VM1 Application System (version 1.0.7.61b, Motic, Xiamen, China).

### 2.5. Determination of the Cd Content in the Liver Tissue, Serum, and Feces

The liver tissue and feces (100 mg), as well as serum (100 μL), were put in a microwave digestion tank with an added 5 mL of nitric acid before being pre-digested at 120 °C for 25 min, digested at 150 °C for 2 h, and then digested at 180 °C until the sample was colorless. We then set the volume to 50 mL with water before filtering and testing with inductively coupled plasma–mass spectrometry.

### 2.6. Real-Time Quantitative Polymerase Chain Reaction (RT-qPCR)

The VeZol-Pure Total RNA lsolation Kit (Cat RC202-01, Vazyme Biotech, Nanjing, China) was used to extract RNA from mice livers. The cDNA was synthesized by Evo M-MLV RT Kit with gDNA Clean for qPCR II (Cat AG11711, Accurate, Changsha, China). SYBR Green Premix Pro Taq HS qPCR Kit (Cat AG11701, Accurate, Changsha, China) was used for RT-qPCR. RT-qPCR was performed on an QuantStudio 5 Real-Time PCR System (Thermo Fisher Scientific, Waltham, MA, USA). The RT-qPCR procedure was as follows: pre-denaturation at 95 °C for 30 s followed by a total of 40 cycles of denaturation at 95 °C for 5 s, with an extension at 60 °C for 30 s, and then melting at 95 °C for 15 s, 60 °C for 1 min, and 95 °C for 1 s [35,36]. The primer sequences (Supplementary Table S2) were synthesized by Tsingke Biotech Co., Ltd. (Beijing, China). The mRNA levels of each target were calculated using the ΔΔCT method relative to the reference gene, GAPDH.

### 2.7. Western Blot Assay

The protein of mice livers were extracted with RIPA buffer (high) (Cat R0010, Solarbio, Beijing, China). Protein concentrations were determined with the BCA protein assay kit (Cat PC0020 Solarbio, Beijing, China). Proteins were separated by SDS-PAGE, transferred to PVDF membranes (Millipore, Bedford, MA, USA), and sealed with 5% skimmed dry milk at room temperature for 2 h. The membrane was incubated separately with primary antibodies of SIRT1 (Cat PQA2569, Abmart, Shanghai, China), alpha-smooth muscle actin (*α*-SMA) (Cat, bs-10196R, Bioss, Beijing, China), *alpha* 1 type I collagen (Col1*a*1) (Cat, bs-10423R, Bioss), transforming growth factor *beta*1 (TGF *β*1) (Cat, 21898-1-AP, Proteintech), nuclear factor erythroid 2-related factor 2 (Nrf2) (Cat 16396-1-AP, Proteintech), *β*-Actin (Cat 10094-1-AP, Proteintech), peroxisome proliferator-activated receptor gamma coactivator 1*α* (PGC-1*α*) (Cat, 66369-1-Ig, Proteintech), Parkin (Cat, 14060-1-AP, Proteintech), insulin receptor (INSR) (Cat, 20433-1-AP Proteintech), protein kinase B (AKT) (Cat, 60203-2-Ig), phosphorylation AKT (Ser473) (*p*-AKT (Ser473)) (Cat 66444-1-Ig, Proteintech), Sterol regulatory element-binding transcription factor 1 (SREBP1) (Cat 14088-1-AP, Proteintech), fatty acid synthase (FASN) (Cat 81079-1-RR, Proteintech), carnitine palmitoyltransferase 1 *alpha* (CPT1*α*) (Cat 15184-1-AP, Proteintech), peroxisome proliferator-activated receptor *alpha* (PPAR*α*) (Cat 66826-1-Ig, Proteintech), Peroxisomal acyl-Coenzyme A oxidase 1 (ACOX1) (Cat 83731-2-RR, Proteintech) and glucose transporter 2 (GLUT2) (Cat 20436-1-AP, Proteintech), *p*-mTOR (Ser2481) (Cat 28879-1-AP, Proteintech), mTOR (Cat 28273-1-AP, Proteintech), glutathione peroxidase 4 (GPX4) (Cat 67763-1-Ig, Proteintech), ferroptosis suppressor protein 1 (FSP1) (Cat 20886-1-AP, Proteintech), acyl-CoA synthetase long-chain family member 4 (ACSL4) (Cat 22401-1-AP, Proteintech), Ubiquitin (Cat 10201-2-AP, Proteintech) (Wuhan, China), phosphoinositide-3-kinase (PI3K) (Cat 4249T, Cell Signaling), phosphorylation PI3K p85 (Tyr458)p55 (Tyr199) (pPI3Kp85 (Tyr458)p55 (Tyr199)) (Cat 17366T, Cell Signaling), *p*-AMPK*α* (Thr172) (Cat 2535T, Cell Signaling), AMPK*α* (Cat 5831T, Cell Signaling), ribosomal protein S6 kinase beta-1 (P70S6K) (Cat 34475T, Cell Signaling), *p*-P70S6K (Ser371) (Cat 9208T, Cell Signaling), Acetyl-CoA carboxylase (ACC) (Cat 3676T, Cell Signaling), phosphorylation ACC (*p*-ACC (Ser79)) (Cat 11818T, Cell Signaling) (Danvers, MA, USA) at 4 °C for overnight, and incubated with Dylight 800-Goat Anti-Rabbit IgG secondary antibody (Cat A23920, Abbkine, Wuhan, China) at room temperature for 1 h [36,37]. The membranes were then scanned by an Odyssey CLx Imaging System (LI-COR Biosciences, Lincoln, NE, USA). The grayscale analyses for protein bands were carried out using Image J Software (version 1.53n).

### 2.8. Statistical Analysis

The results are the means ± SEM. Statistical differences between groups were evaluated by or *t*-test, as appropriate. For comparisons among multiple groups, one-way analysis of variance (ANOVA) was used. When ANOVA indicated a significant difference (*p* < 0.05), Tukey’s multiple comparison test/Dunnett’s multiple comparison test/Games–Howell post hoc test was performed. For comparisons between two groups, an unpaired two-tailed Student’s *t*-test was used; if variances were unequal, Welch’s *t*-test was applied. Non-normally distributed data were analyzed using the Kruskal–Wallis test followed by Dunn’s multiple comparison test. A two-sided *p* value < 0.05 was considered statistically significant. GraphPad Prism software (version 9.5.0, San Diego, CA, USA) was used for statistical analysis. The exact number of mice (n) used for each analysis is specified in the corresponding figure and table legends.

## 3. Results

### 3.1. LT Significantly Reduces the Mortality Caused by Acute Cd Exposure in Mice

**Figure 1:**
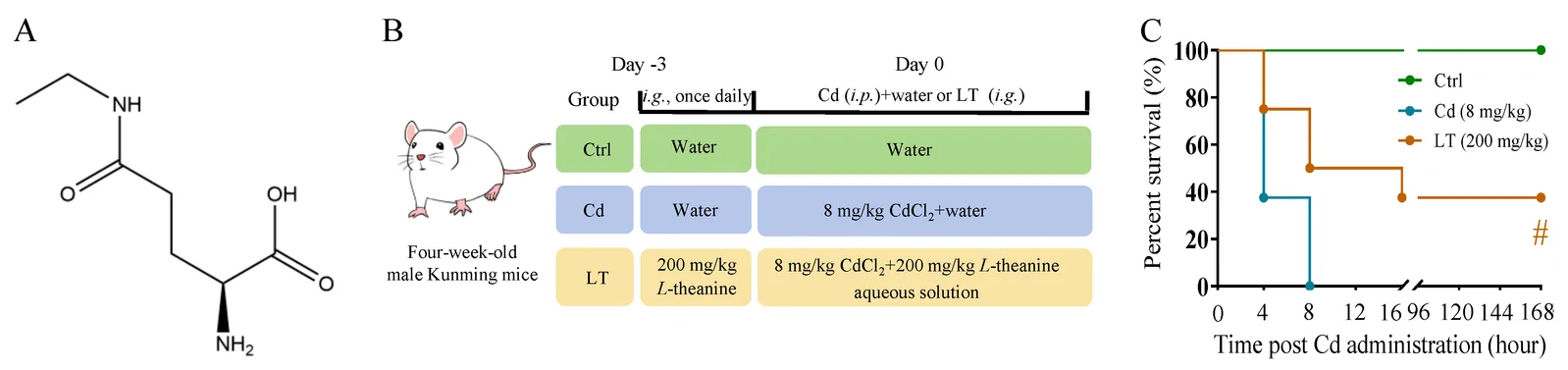
Effects of LT on mortality in mice with acute exposure to Cd. (**A**) Chemical structure of LT. (**B**) Schematic diagram of animal experiment. (**C**) Survival time post Cd treatment (n = 8). # *p* < 0.05, compared to Cd group.

To investigate the protective potential of LT on the acute toxicity of Cd exposure, we first examined the effect of LT on survival time in acute Cd-exposed mice. The results showed that a single intraperitoneal injection of Cd (8 mg/kg) caused 60% mortality within 4 h and 100% mortality within 8 h in mice. Continuously intragastric administration of LT at a dose of 200 mg/kg for 4 days reduced the mortality of mice from 60% to 30% at 4 h and from 100% to 50% at 8 h, significantly prolonged the median survival time of mice from 4 h to 16 h after acute Cd exposure and reduced overall mortality to 60% (Figure 1B,C).

### 3.2. LT Ameliorates Liver Dysfunction Caused by Subchronic Cd Exposure in Mice

**Figure 2:**
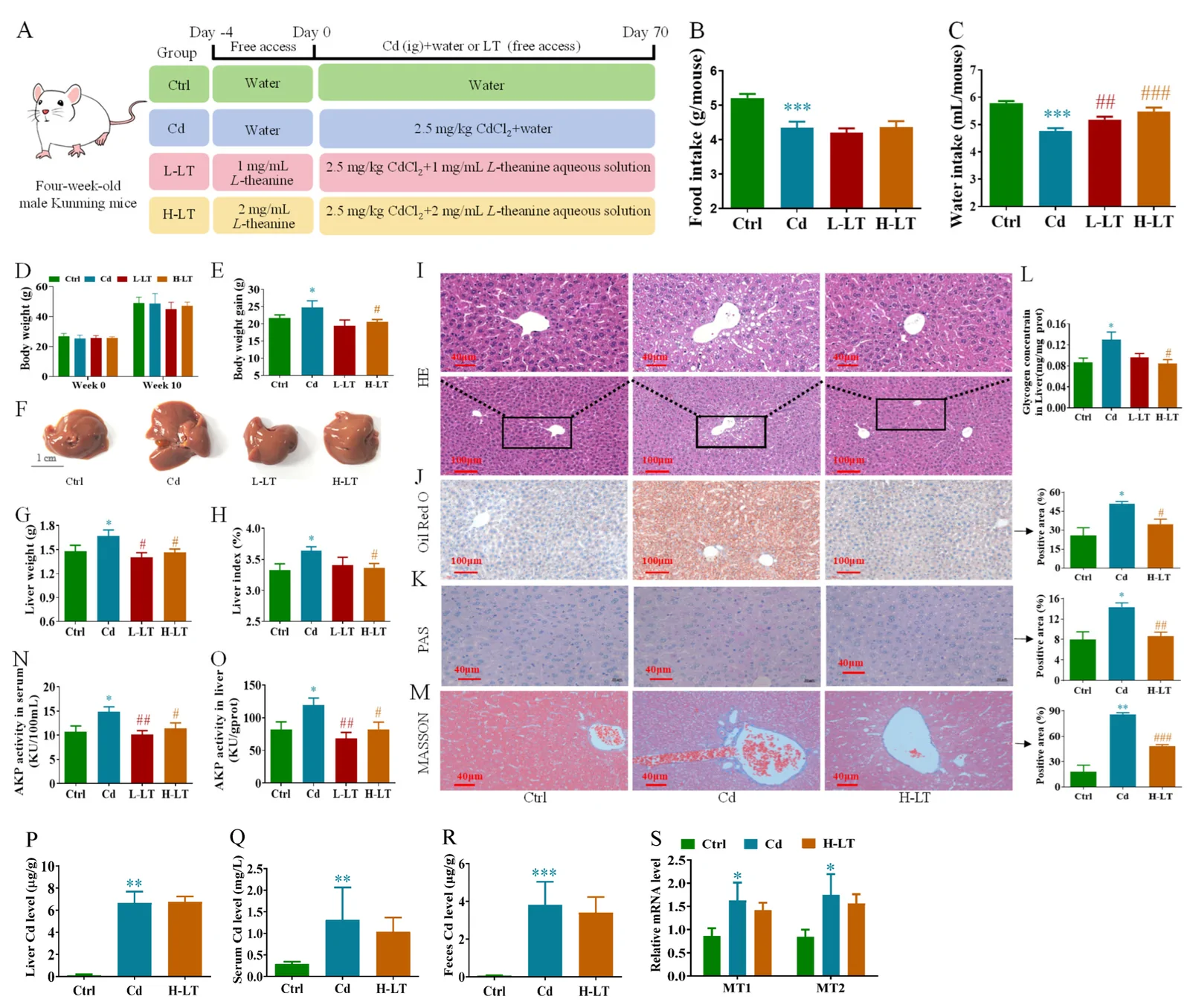
Effects of LT on body weight, liver histology, and function in mice with subchronic exposure to Cd. (**A**) Schematic diagram of animal experiment. (**B**,**C**) Food and fluid intakes. (**D**,**E**) Body weight and body weight gain. (**F**) Liver morphology. (**G**) Liver weight. (**H**) Liver index. (**I**–**K**) H&E, Oil Red O, and Masson staining. (**L**,**M**) Glycogen levels and PAS staining. (**N**,**O**) AKP activity levels in serum and liver, respectively. (**P**–**R**) Cd levels in liver, serum, and feces, respectively. (**S**) Relative mRNA expression levels of *MT1* and *MT2*. Data are presented as the mean ± SEM (n = 9). * *p* < 0.05, ** *p* < 0.01 and *** *p* < 0.001, compared to Ctrl group. # *p* < 0.05, ## *p* < 0.01 and ### *p* < 0.001, compared to Cd group.

The alleviating effects of LT on liver function in mice subjected to subchronic Cd exposure were investigated next. The results showed that a ten-week Cd exposure significantly decreased food and fluid intake without affecting the body weight of mice, although it did increase body weight gain. The administration of both doses of LT elevated fluid intake, but it did not affect food intake and body weight. Notably, the high dose of LT effectively inhibited the increase in body weight gain (Figure 2A–E). Severe liver hypertrophy, fatty infiltration and amyloidosis, and the accumulation of lipid and glycogen (Figure 2D) were observed as a result of Cd exposure, as indicated by the liver morphology, weight, and index through Oil Red O, PAS, and H&E staining, respectively (Figure 2G–L, Table 1). In addition, Masson staining showed collagen accumulation due to Cd exposure, suggesting fibrotic lesions and liver damage (Figure 2M). This was corroborated by significantly increased activity levels of AKP in both serum and the liver (Figure 2N,O). LT treatment effectively prevented or improved these lesions (Figure 2F–O). The observed impairments following Cd exposure could be attributed to markedly elevated Cd levels in the liver and serum. Cd exposure also increased Cd levels in the feces and *metallothionein* (*MT*) *1* and *MT2* mRNA levels in liver. However, LT neither affects the accumulation of Cd in these samples nor influences the mRNA levels of *MT* in the liver (Figure 2P–S).

### 3.3. LT Improves Mitochondrial Dysfunction Caused by Subchronic Cd Exposure in Mice by Regulating the SIRT1/PGC-1α Signaling Pathway 

**Figure 3:**
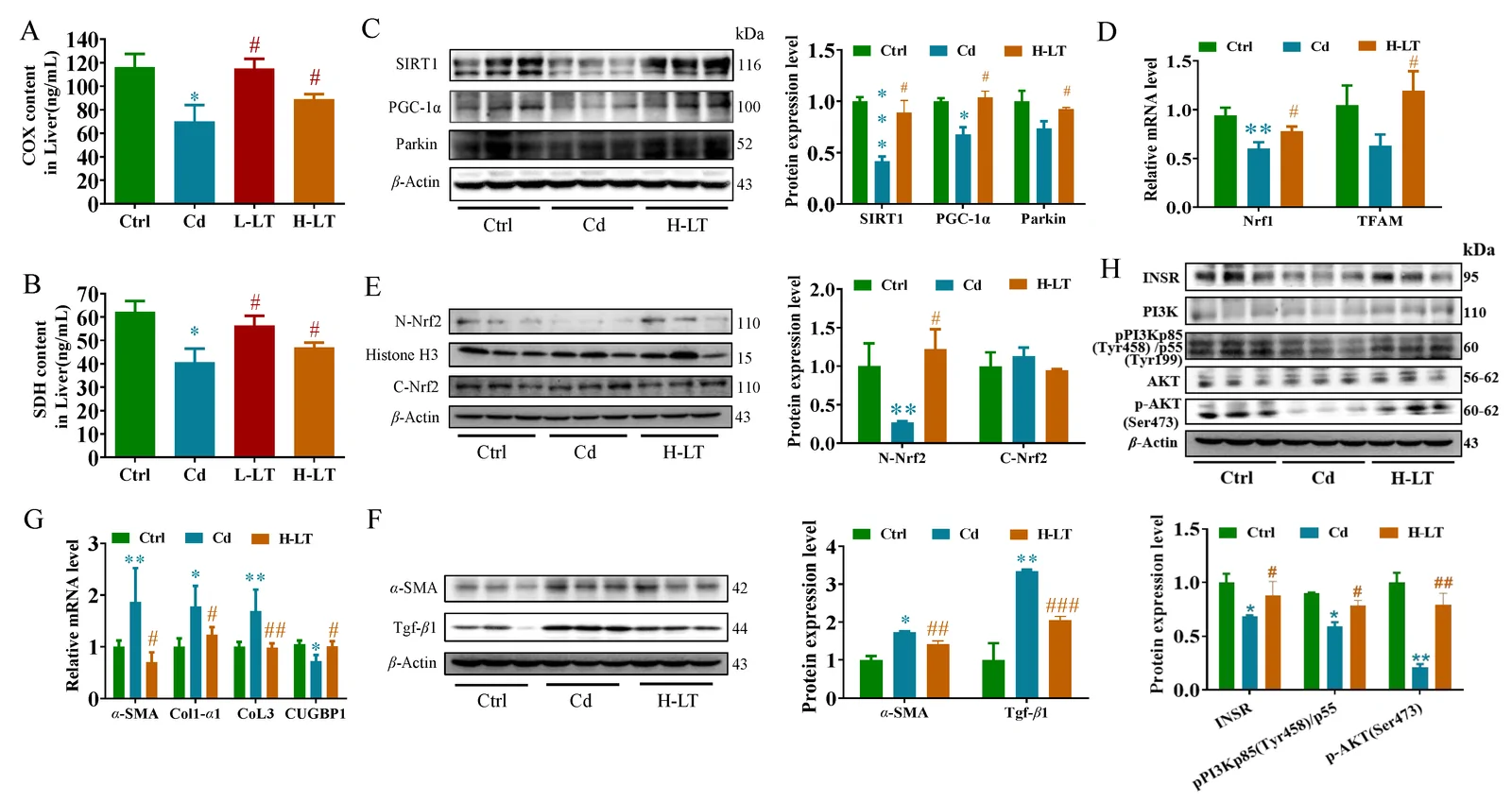
Effects of LT on mitochondrial function, SIRT1/PGC-1*α* signaling pathway, and its downstream targets in the liver of subchronic Cd-exposed mice. (**A**,**B**) COX and SDH levels (n = 9). (**C**) SIRT1/PGC-1*α* signaling pathway associated proteins (n = 3). (**D**) Relative mRNA expression levels of *TFAM* and *NRF1* (n = 9). (**E**) Nrf2 protein expression. (**F**,**G**) Fibrosis-related proteins and genes. (**H**) Insulin signaling pathway proteins (n = 3). The displayed blots were cropped from original full-length membranes, and we have added a statement in the Original Images for Blots or Gels file describing the membrane cutting procedure. Data are presented as the mean ± SEM. * *p* < 0.05, ** *p* < 0.01 and *p* < 0.001, compared to Ctrl group. # *p* < 0.05, ## *p* < 0.01 and ### *p* < 0.001, compared to Cd group.

Heavy-metal exposure is known to induce mitochondrial dysfunction, disrupt energy metabolism, and cause oxidative stress. Indeed, this study found that subchronic Cd exposure significantly reduced the levels of key hepatic mitochondrial function markers, including COX and SDH (Figure 3A,B). At the molecular level, Cd downregulated the expression of proteins central to mitochondrial biogenesis and quality control, such as SIRT1, PGC-1*α*, and Parkin proteins, as well as *nuclear respiratory factor 1* (*Nrf1*) and *mitochondrial transcription factor A* (*TFAM*) genes (Figure 3C,D). These findings indicate that Cd exposure impairs mitochondrial function and suppresses mitophagy. This was further supported by the downregulation of the downstream N-Nrf2, the upregulation of pro-fibrogenic genes (*α-SMA*, *Col1α1*, *Col3*) and proteins (*α*-SMA, TGF-*β*1), and the downregulation of the anti-fibrogenic gene *CUGBP1* (Figure 3E–G). Importantly, intervention with LT effectively prevented these alterations (Figure 3). In addition, we found that the insulin signaling pathway mediated by SIRT1 was suppressed by Cd exposure, as indicated by the significantly downregulated NISR, *p*-PI3Kp85 (Tyr458)p55 (Tyr199), and *p*-AKT (Ser473) proteins. LT treatment prevented the downregulation of insulin signaling pathway (Figure 3H). These results suggest LT can alleviate mitochondrial dysfunction caused by subchronic Cd exposure.

### 3.4. LT Alleviates Ferroptosis in Liver Caused by Subchronic Cd Exposure in Mice

**Figure 4:**
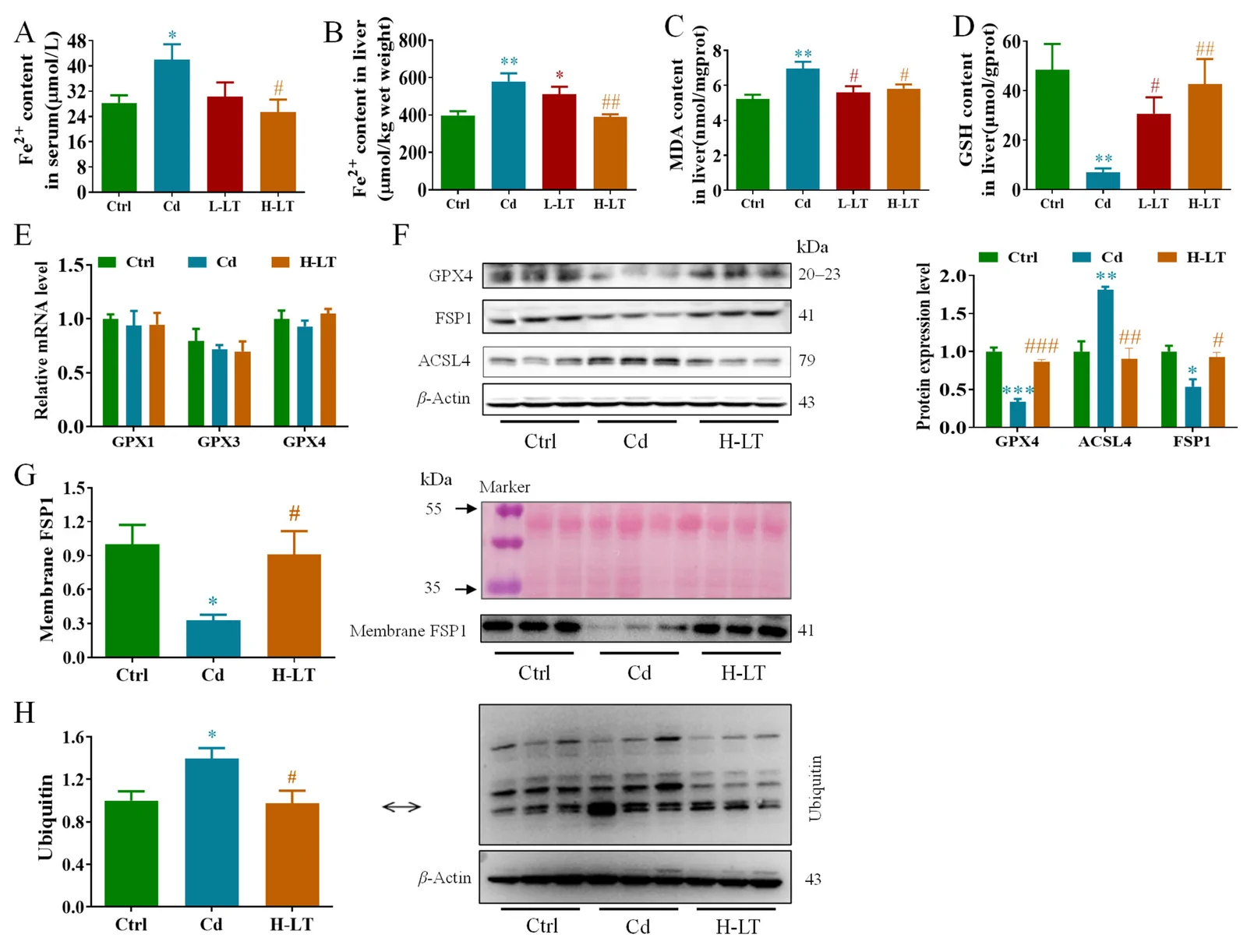
Effects of LT on ferroptosis in the liver of subchronic Cd-exposed mice. (**A**,**B**) Levels of Fe^2+^ in serum and liver. (**C**,**D**) Levels of MDA and GSH. (**E**) Relative mRNA levels of *GPX1*, *GPX3*, and *GPX4* (n = 9). (**F**) GPX4, FSP1, and ACSL4 protein expression. (**G**) Membrane FSP1 protein expression. (**H**) Ubiquitin protein expression (n = 3). The displayed blots were cropped from the original full-length membranes, and we have added a statement in the Original Images for Blots or Gels file describing the membrane cutting procedure. Data are presented as the mean ± SEM. * *p* < 0.05, ** *p* < 0.01 and *** *p* < 0.001, compared to Ctrl group. # *p* < 0.05, ## *p* < 0.01 and ### *p* < 0.001, compared to Cd group.

We next examined the impacts of Cd exposure on key ferroptotic biomarkers. The results showed that Cd exposure significantly elevated Fe^2+^ levels in both serum and the liver; increased hepatic MDA, a lipid peroxidation product; and decreased the ferroptosis inhibitor GSH (Figure 4A–D). At the molecular level, although Cd exposure did not affect the mRNA levels of *GPX1*, *3*, and *4*, it downregulated the key anti-ferroptotic proteins, including GPX4, FSP1, and membrane FSP1, while upregulating the pro-ferroptotic protein ACSL4 (Figure 4E–G) and markedly enhancing protein ubiquitination (Figure 4H). These findings indicate that subchronic Cd exposure triggers ferroptosis by promoting the ubiquitination-mediated degradation of GPX4. Considering the involvement of AMPK-mediated *p*-ACC (Ser79) in the regulation of ferroptosis, and AMPK/mTOR signaling pathway in the regulation of autophagy, the modulating effects of Cd exposure on *p*-AMPK (Thr172)/*p*-ACC (Ser79) and AMPK/mTOR signaling pathways were subsequently investigated to further determine whether subchronic Cd exposure induces ferroptosis and autophagic response disorder.

### 3.5. LT Blocks Autophagy Inhibition in Liver Caused by Subchronic Cd Exposure by Upregulating the AMPK/mTOR Signaling Pathway in Mice

**Figure 5:**
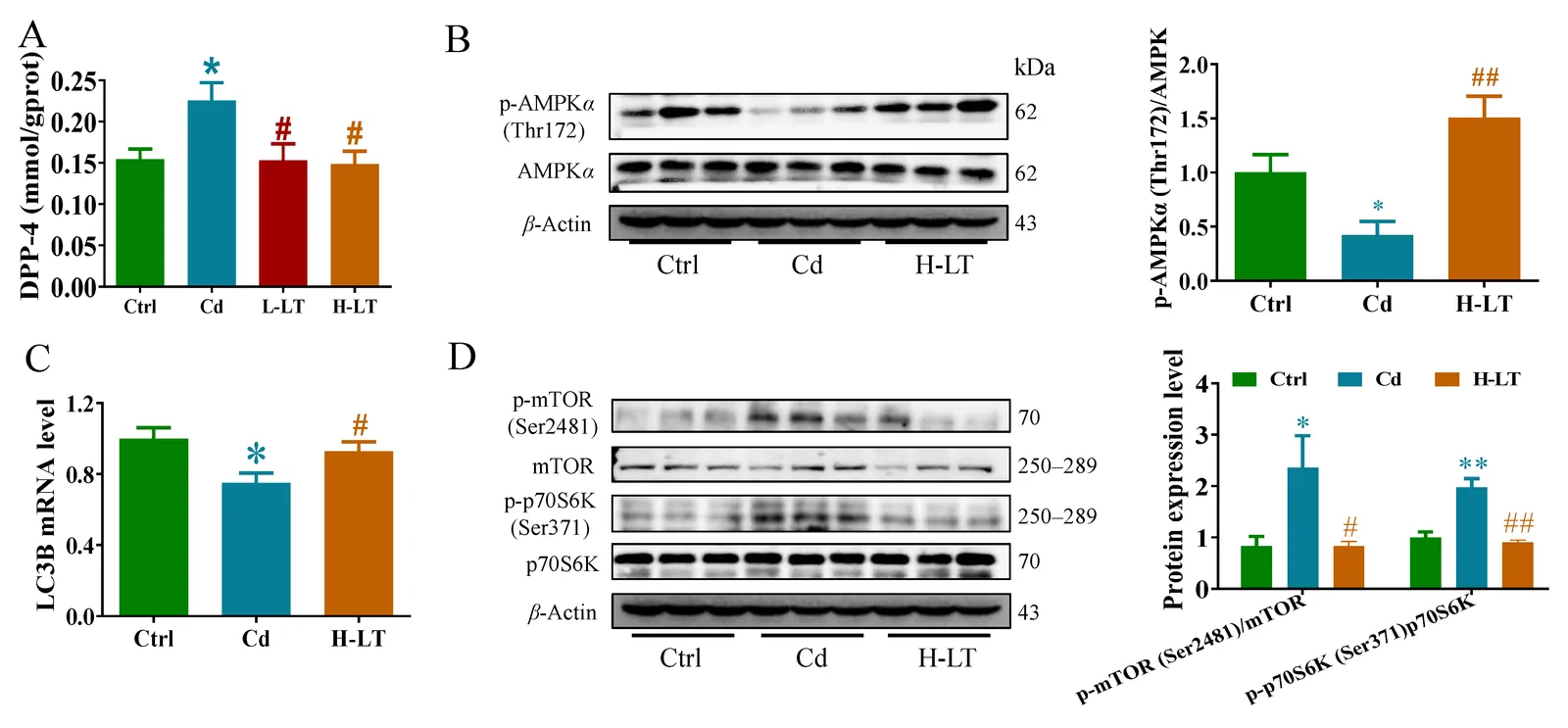

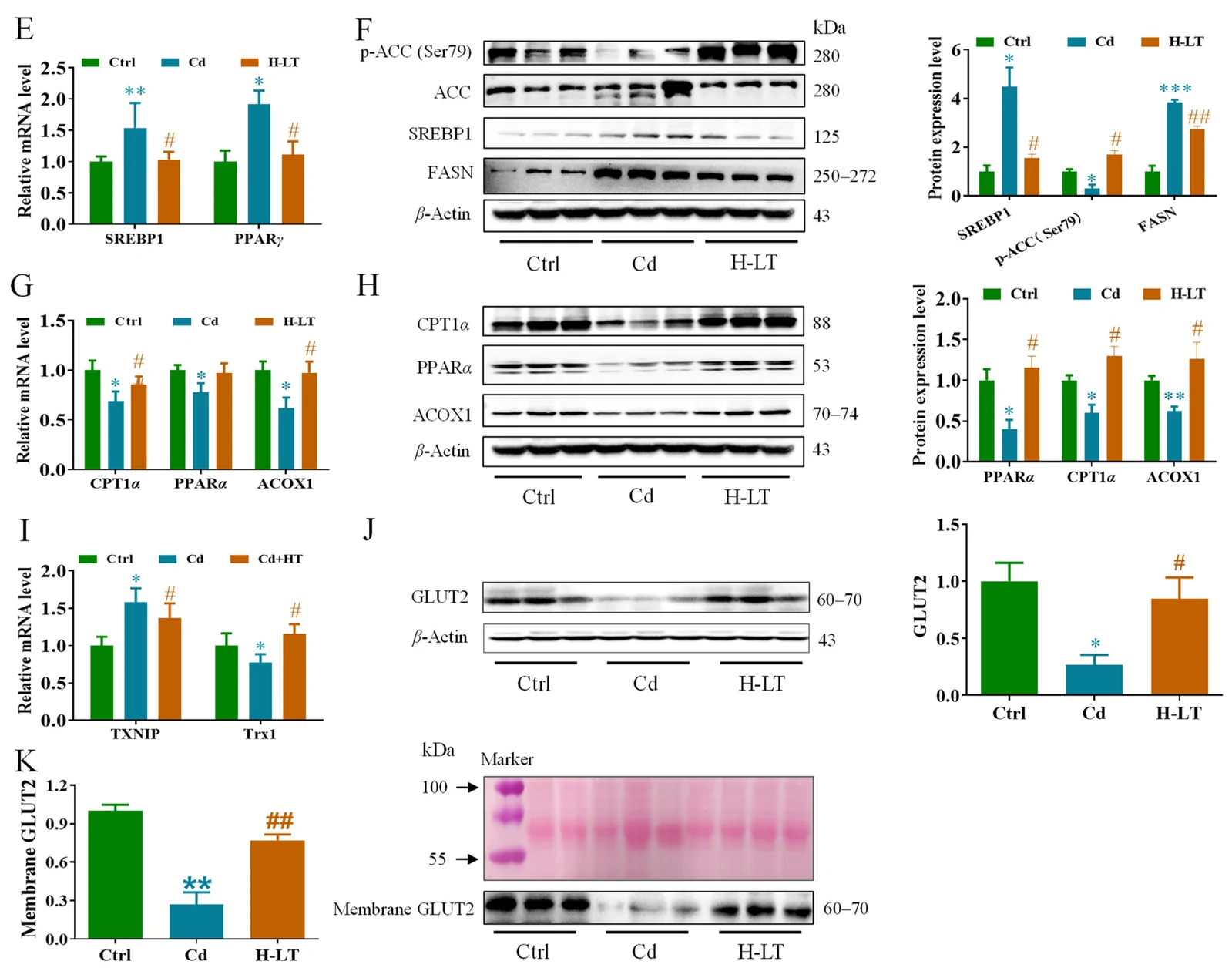
Effects of LT on AMPK/mTOR-mediated autophagy signaling pathway and AMPK downstream glucolipid metabolism-associated genes and proteins in the liver of subchronic Cd-exposed mice. (**A**) DPP-4 level (n = 9). (**B**) *p*-AMPK (Thr172) protein expression (n = 3). (**C**) Relative mRNA levels of *LC3B* (n = 9). (**D**) *p*-mTOR (Ser2481) and *p*-p70S6K (Ser371) proteins expression (n = 3). (**E**,**F**) Fatty acid synthesis-related genes (n = 9) and proteins (n = 3). (**G**,**H**) Fatty acid oxidation related genes (n = 9) and proteins (n = 3). (**I**) Relative mRNA levels of gluconeogenesis genes *TXNIP* and *Trx1* (n = 9). (**J**,**K**) Glucose transporter GLUT2 and membrane GLUT2 expression (n = 3). The displayed blots were cropped from the original full-length membranes, and we have added a statement in the Original Images for Blots or Gels file describing the membrane cutting procedure. Data are presented as the mean ± SEM. * *p* < 0.05, ** *p* < 0.01 and *** *p* < 0.001, compared to Ctrl group. # *p* < 0.05 and ## *p* < 0.01 compared to Cd group.

Subchronic exposure to Cd resulted in an increase in hepatic levels of DPP-4, which acts as a negative regulator of AMPK, thereby causing a downregulation of phosphorylated AMPK at threonine 172 (Figure 5A,B). The suppression of *p*-AMPK (Thr172) subsequently led to an upregulation of autophagy-inhibitory proteins, specifically *p*-mTOR (Ser2481) and *p*-p70S6K (Thr389), as well as an increase in microtubule-associated protein LC3B (Figure 5C,D). As a consequence of *p*-AMPK (Thr172) inhibition, there was a significant downregulation of critical downstream targets, including *p*-ACC (Ser79), markers of fatty acid oxidation (CPT1*α*, PPAR*α*, ACOX1), as well as the glucose transporter GLUT-2 and membrane GLUT-2. In contrast, there was a marked upregulation of lipogenic genes and proteins, including SREBP1, FASN, and PPAR*γ*, along with the gluconeogenic gene *thioredoxin-interacting protein* (*TXNIP*) (Figure 5E–K). These results demonstrate that subchronic Cd exposure impairs hepatic autophagy and disrupts glucolipid metabolic homeostasis by suppressing the AMPK/mTOR signaling pathway and its downstream metabolic targets (Figure 5). Treatment with LT effectively counteracted the suppression of the AMPK/mTOR pathway, and these effects were accompanied by findings consistent with modulation of autophagy-related signaling and by maintenance glucolipid metabolic homeostasis (Figure 5). Moreover, the significant downregulation of both *p*-AMPK (Thr172) and *p*-ACC (Ser79) induced by Cd exposure suggests the occurrence of ferroptosis, which was effectively mitigated by LT intervention (Figure 5B,F). This findings suggest that subchronic Cd exposure co-induces ferroptosis by concomitantly modulating the *p*-AMPK/*p*-ACC axis (Figure 5B,F) and the canonical ferroptosis pathway (Figure 4).

## 4. Discussion

MT, a low-molecular-weight protein, has shown a high affinity with metal ions and a strong free radical scavenging activity, and it can efficiently bind with metal ions at a molecular ratio of 1:7–12 to facilitate the conversion of free heavy-metal ions to low-toxicity heavy-metal ion–MT complexes, thereby antagonizing Cd toxicity [38,39]. Reducing Cd accumulation in tissues and serum via promoting Cd excretion by chelation and upregulating MT expression in tissues is considered one of the most effectively and direct approaches for preventing Cd toxicity [40,41]. LT can mitigate cytotoxicity caused by copper-facilitated dopamine and (-)-epigallocatechin-3-gallate oxidation by chelating copper ions, which is an important mechanism through which LT reduces copper toxicity. However, to the best of our knowledge, no study has reported that LT possesses a chelating capacity for Cd. In this study, we observed that Cd exposure markedly elevated Cd levels in the liver, serum, and feces, and significantly upregulated the mRNA expression levels of *MT1* and *MT2* genes in the liver. These changes may represent an adaptive protection mechanism. LT treatment did not affect the accumulation levels of Cd in the liver, serum, and feces, nor did it influence the mRNA expression levels of *MT1* and *MT2* genes in the liver. These results suggest that the ameliorative effects of LT on liver dysfunction caused by Cd exposure may be attributed to a unique mechanism distinct from the mechanisms mentioned above.

Autophagy, an evolutionarily conserved self-digestive process, is considered as a critical cell survival mechanism. Heavy metals impair autophagy flux and inhibit lysosomal degradation capacity, leading to cellular renovation failure [42]. Key regulators of autophagy include PI3K, AMPK and the autophagy inhibitor mTOR [43]. mTOR negatively regulates autophagy, as its activation is associated with the upregulation of the PI3K/AKT pathway and the downregulation of AMPK. AMPK promotes autophagy by dissociating from the Atg2/unc-51-like kinase complexes (ULK) and dephosphorylating ULK1 and ULK2, thereby inhibiting the formation of multiprotein kinase complex 1 of mTOR [43]. We found that Cd exposure inhibits autophagy, as evidenced by the downregulated *LC3B* and upregulated phosphorylated mTOR (Ser2481) and phosphorylated p70S6K (Ser371), a target of mTOR [44], which may be attributed to the downregulation of upstream phosphorylated AMPK(Thr172). The study by Song et al. [44] demonstrated that sitagliptin, a selective DPP-4 inhibitor, effectively counteracted the downregulation of AMPK and upregulation of mTOR, as well as the inhibition of autophagy induced by Cd exposure. This finding suggests a critical role for DPP-4 in the regulation of autophagy via the AMPK/mTOR pathway. This hypothesis is further supported by our study, which observed a significant increase in DPP-4 levels in the liver following Cd exposure. Additionally, evidence indicates that excessive exposure to heavy metals downregulates SIRT1 expression and activity, consequently diminishing the deacetylation rate of its target proteins, such as LKB1 [14]. A lower rate of LKB1 deacetylation contributes to the metal-induced reduction in AMPK activity and subsequent metabolic dysfunctions [14]. These reports and our results suggest that the DPP-4/AMPK and SIRT1/LKB1/AMPK axes are involved in the inhibition of autophagy mediated by the mTOR/p70S6K activation due to Cd exposure. Furthermore, LT treatment effectively prevented the downregulation of AMPK and the activation of mTOR/p70S6K by reducing DPP-4 levels and upregulating SIRT1 in the liver, thereby preventing autophagy inhibition caused by Cd exposure. Though the upregulation of *LC3B* mRNA, together with the observed downregulation of phosphorylated mTOR (Ser2481) and phosphorylated p70S6K (Ser371), is consistent with changes in autophagy-related signaling following LT treatment, the current data do not directly demonstrate autophagy activation. Moreover, an important limitation that should be emphasized is that the present data cannot distinguish between increased autophagosome formation and impaired autophagic degradation, and that future protein-level and flux analyses are required.

Epidemiological studies have demonstrated a strong positive correlation between serum Cd levels and the incidence of various chronic liver diseases, such as liver steatosis [45]. Both existing reports and the present study showed that Cd exposure leads to the downregulation of AMPK [12,14,44] and the PI3K/AKT signaling pathways, which are critical targets for maintaining energy homeostasis and may contribute to the pathogenesis of these chronic liver diseases. AMPK serves as a critical sensor of cellular energy status and is activated via AMP binding and upstream kinase phosphorylation in response to energy stress [46]. Once activated, AMPK can restore energy balance and maintain cell survival during energy stress by phosphorylating a myriad of downstream targets, hereby enhancing ATP-generating catabolic processes while inhibiting ATP consuming anabolic processes [47]. The PI3K/AKT axis, a pivotal component of the insulin pathway, plays a crucial role in regulating hepatic glycogen synthesis, gluconeogenesis, and lipid synthesis. Activation of PI3K/AKT signaling pathway has been shown to ameliorate glucolipid dysmetabolism. Improving insulin sensitivity by upregulating the PI3K/AKT axis is considered a promising strategy, as PI3K facilitates insulin actions by transferring signals from insulin receptors (INSRs) to downstream targets [48,49]. In this study, we observed that Cd exposure led to dysregulated glucose and lipid metabolism, as evidenced by significantly elevated levels of Cd and TG in serum and TC in the liver, along with decreased levels of HDL-C in both serum and the liver. Furthermore, Cd exposure resulted in the downregulation of the NISR/PI3K/AKT and AMPK signaling pathways. The observed increase in lipid accumulation due to Cd exposure may be attributed to the downregulation of CPT1*α*, PPAR*α*, ACOX1, and *p*-ACC (Ser79), as well as the upregulation of SERBP1, FASN, and PPAR*γ*, which are downstream targets of AMPK involved in fatty acid oxidation or lipid synthesis. The observed elevation in liver glycogen levels can be attributed to the downregulation of GLUT2 and thioredoxin (Trx), alongside the upregulation of TXNIP. Importantly, LT intervention improved hepatic steatosis caused by Cd exposure by preventing the downregulation of the SIRT1/NISR/PI3K/AKT axis and the AMPK signaling pathway.

Mitochondria, as the primary sites for ATP and ROS production, play a crucial role in maintaining redox and energy homeostasis. It has been demonstrated that Cd can inhibit the expression of SIRT1 and SIRT3 [14]. The downregulation of SIRT subsequently suppress the deacetylation of target proteins, leading to increased acetylation that downregulates PGC1*α*, a known target of SIRT1 [50]. This results in the inhibition of antioxidant defense system and decreased mitochondrial biogenesis [14]. Our results suggest that Cd exposure leads to structural and functional abnormalities in mitochondria, as indicated by the suppressed activity levels of mitochondrial function markers COX and SDH. These abnormalities may be attributed to the downregulation of the SIRT1/PGC1*α*/Nrf2/TFAM and SIRT1/PGC1*α*/Nrf1/TFAM signaling pathways. As a consequence of mitochondrial dysfunction caused by Cd exposure, liver fibrosis was observed, as indicated by the accumulated collagen and upregulated *α*-SMA and TGF-*β*1. LT treatment effectively restored mitochondrial function and reduced collagen accumulation by upregulating the SIRT1/PGC1*α*/Nrfs/TFAM signaling pathway.

Ferroptosis, a recently identified form of regulated necrotic cell death, is primarily driven by iron-dependent lipid peroxidation, particularly of phospholipids. The activation of AMPK under conditions of energy stress inhibits ferroptosis by phosphorylation of ACC at serine 79 (ACC1) or serine 212 (ACC2) site, thereby obstructing the biosynthesis of polyunsaturated fatty acids (PUFAs) [51]. Lipid peroxidation is predominantly induced by the excessive production of a specific class of ROS, known as phospholipid peroxides (PLOOH), in the process of cellular metabolism driven by oxygen and iron. This process preferentially targets PUFAs, especially PUFA-containing phospholipids (PUFA-PLs) [52]. Because PUFA-PLs are integral structural components of the lipid bilayers in cell membranes, their peroxidation compromises membrane integrity, leading to cellular damage and the generation of secondary toxic products such as 4-HNE and MDA, ultimately culminating in ferroptosis [51,53]. ACSL4 and lysophosphatidylcholine acyltransferase 3 facilitate the activation of long-chain PUFAs, contributing to membrane phospholipid synthesis, this process results in the accumulation of PUFA-PLs and subsequently initiates ferroptosis. Moreover, ferroptosis is modulated by the GSH redox system. The depletion of GSH leads to the inactivation of GPX4 and the accumulation of PLOOH [54]. GPX4 is the sole mammalian enzyme capable of catalyzing the reduction in phospholipid hydroperoxides to phospholipid alcohols, thereby inhibiting enzymes involved in arachidonic acid (AA) metabolism, which is the most significant PUFA. This enzyme plays a crucial role in reducing lipid peroxidation and blocking ferroptosis [52,55].

Certain enzymes, such as FSP1, GTP cyclohydrolase 1, and dihydroorotate dehydrogenase, have the capacity to terminate the Fenton radical chain reaction and prevent the amplification of lipid peroxidation by producing metabolites with free-radical-trapping properties; these enzymes are considered to be GPX4-independent mechanisms that can mitigate ferroptosis [52,56]. In the context of Cd exposure, ferroptosis is indicated by significantly elevated levels of Fe^2+^ both in serum and liver, as well as increased levels of MDA and ACSL4 proteins in the liver. Concurrently, there is a marked reduction in the levels of GSH, *p*-AMPK (Thr172), *p*-ACC (Ser79), GPX4, FSP1, and membrane FSP1 proteins in the liver. LT treatment effectively counteracts these alterations and prevents ferroptosis. The underlying mechanism involves the upregulating *p*-AMPK (Thr172) and *p*-ACC (Ser79) to block PUFA biosynthesis, the trafficking of FSP1 to the apical plasma membrane to prevent phospholipid peroxidation, and the reduction in GPX4 ubiquitination degradation to maintain the transformation of phospholipid hydroperoxides into low-toxic phospholipid alcohols, thereby reducing AA metabolism. A limitation worth noting is that the Cd-induced increase in total ubiquitination, coupled with decreased GPX4 protein expression, is consistent with, but does not prove, GPX4 ubiquitination. Co-immunoprecipitation with GPX4 antibody followed by anti-ubiquitin blotting is required for definitive confirmation, which we plan for future studies.

Although the present study is primarily based on a rodent model, accumulating evidence supports the potential involvement of Cd-induced ferroptosis in human hepatic pathology. For instance, in vitro studies using human hepatocyte cell lines have verified that Cd exposure triggers canonical ferroptotic signals mediated by the ACSL4/GPX4 axis [57]. Epidemiological investigations have revealed positive correlations between blood Cd concentrations and circulating levels of lipid peroxidation end-products (e.g., MDA and 4-HNE) in exposed populations, indicating that systemic oxidative stress and phospholipid peroxidation also occur in humans [58]. Moreover, a re-analysis of public transcriptomic datasets from heavy-metal-induced liver injuries has shown significant enrichment of the ferroptosis pathway. Importantly, the AMPK-ACC-PUFA and GPX4-GSH-FSP1 axes are evolutionarily highly conserved in mammals, with over 90% homology in core protein sequences between mice and humans [59]. Therefore, our mechanistic findings offer valuable translational insights into human hepatotoxicity induced by environmental pollutants. As a natural dietary compound whose safety and bioactivity have been confirmed in various human cell models, LT acts through a multi-target strategy—enhancing AMPK phosphorylation, promoting FSP1 plasma membrane translocation, and suppressing GPX4 ubiquitination—to counteract ferroptosis, providing a novel theoretical framework for future dietary interventions in populations at high risk of heavy-metal exposure. Nevertheless, extrapolation of our conclusions to humans ultimately warrants further validation using human liver organoids or prospective cohort studies based on specific ferroptosis biomarkers, which will be a key focus of our future research.

AMPK is vital for regulating autophagy [43], mitochondrial homeostasis [60,61], ferroptosis [51], and glucolipid metabolism [46]. Activation of AMPK has been shown to restore impaired mitochondrial function, inhibit ferroptosis, and alleviate hepatic steatosis and T2DM by enhancing autophagy; it also offers protects against alcohol-induced liver injury via enhancing mitophagy [51,61,62,63,64]. Moreover, the downregulation of Nrf2, mediated by the SIRT1/PGC1*α* signaling pathway, leads to GSH depletion and GPX4 inactivation, thereby contributing to mitochondrial injury, oxidative stress and ferroptosis [54]. Impaired mitochondria not only disrupt cellular renovation and energy homeostasis but also selectively promote cystine-starvation-induced ferroptosis [65]. In turn, ferroptosis aggravates mitochondrial dysfunction and oxidative stress [66]. Based on the established literature, we propose that AMPK-mediated restoration of autophagy serves as the apical upstream hub, while the suppression of ferroptosis, the activation of the SIRT1/PGC1*α*/Nrf2 antioxidant axis, and the amelioration of glucolipid metabolism are downstream repercussions of this autophagic recovery. The rationale is as follows: Cd-induced AMPK inactivation primarily suppresses autophagy, leading to the accumulation of dysfunctional mitochondria. These defective mitochondria not only become major sources of ROS and lipid peroxidation substrates, but also selectively sensitize cells to ferroptosis [65]. Consequently, the observed downregulation of SIRT1/PGC1*α*/Nrf2 and depletion of GSH/GPX4 are interpretable as oxidative consequences of impaired mitochondrial quality control, rather than as independent primary targets of Cd or LT. Similarly, energy imbalance and glucolipid dysmetabolism are secondary to the failure of mitochondrial renewal and AMPK-mediated energy sensing. Therefore, when LT activates AMPK, the resultant autophagic flux clears the damaged mitochondria, which sequentially and passively alleviates mitochondrial oxidative injury, curtails ferroptotic phospholipid peroxidation, and restores metabolic homeostasis. This hierarchical perspective clarifies that the effects on SIRT1, Nrf2, ACSL4, and GPX4 are coordinated downstream events, rather than isolated parallel mechanisms.

The DPP4/AMPK/mTOR and SIRT1/LKB1/AMPK pathways are highly conserved across mammalian species [67]. Both AMPK and mTOR are fundamental nutrient-sensing kinases with virtually identical amino acid sequences and regulatory mechanisms between mice and humans [68]. The SIRT1-LKB1-AMPK regulatory axis-in which SIRT1 deacetylates and activates LKB1, which in turn phosphorylates and activates AMPK-has been validated in both murine and human cell systems [68]. Similarly, DPP4 is expressed in human liver tissue and has been implicated in chronic liver diseases including non-alcoholic fatty liver disease and hepatocellular carcinoma. Thus, from a molecular perspective, these pathways are structurally and functionally conserved [69]. While direct evidence linking these specific pathways to Cd-induced hepatic injury in humans remains limited, several lines of supportive evidence exist. Tinkov et al. comprehensively reviewed that Cd and other heavy metals can inhibit SIRT1 activity in mammalian systems, and that reduced LKB1 deacetylation may underlie metal-induced decreases in AMPK activity and subsequent metabolic disturbances [14]. In human renal tubular epithelial cells, Cd has been shown to suppress SIRT1 expression and activity [70]. Additionally, epidemiological studies have associated Cd exposure with metabolic disturbances including insulin resistance and type 2 diabetes, conditions in which AMPK/mTOR signaling dysregulation plays a central role [71]. Furthermore, DPP-4 inhibitors have demonstrated protective effects against Cd-induced organ damage in preclinical models [72], and given that DPP-4 inhibitors are FDA-approved drugs with well-characterized safety profiles in humans, this provides an indirect translational bridge. We acknowledge that our mechanistic findings were obtained in a rodent model, and that direct extrapolation to humans requires caution. However, we believe the translational potential is supported by: (1) the high evolutionary conservation of these core metabolic pathways [68]; (2) the availability of DPP-4 inhibitors as clinically approved drugs that could potentially be repurposed [72]; and (3) the growing epidemiological evidence linking Cd exposure to human metabolic and hepatic diseases [71].

## 5. Conclusions

**Figure 6:**
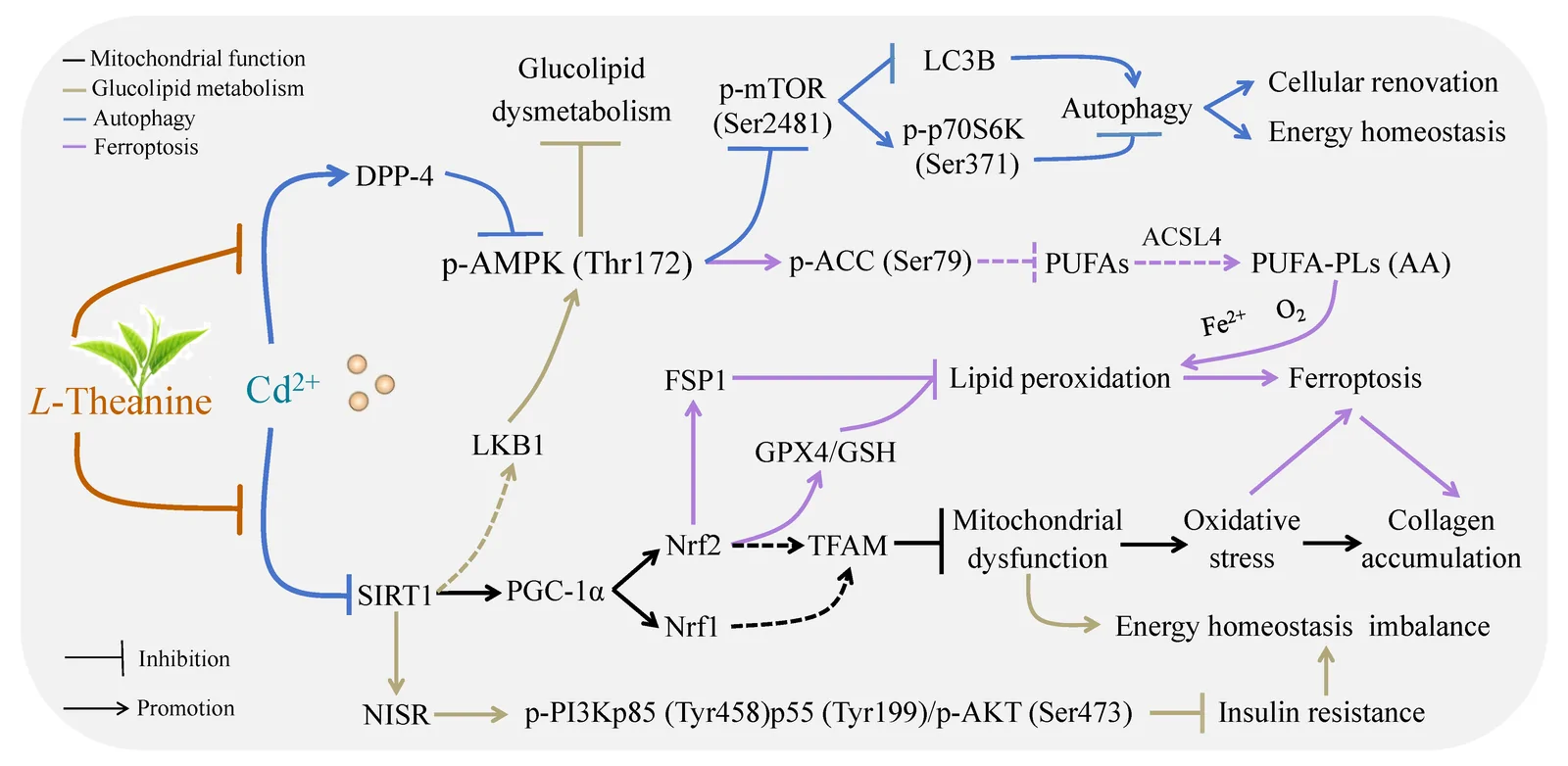
Schematic diagram showing the underlying mechanisms of how LT alleviates Cd-induced mitochondrial dysfunction, ferroptosis, and glucolipid dysmetabolism by promoting autophagy modulated through the DPP4/AMPK/mTOR and SIRT1/AMPK/LKB1/mTOR signaling axes.

Taken together, the major finding of this study is that LT effectively reduced mortality in mice acutely exposed to Cd and mitigated subchronic Cd-induced injury in a Cd bioaccumulation level-independent manner. These effects were accompanied by changes in autophagy-related signaling and by modulation of the DPP-4/AMPK/mTOR and SIRT1/AMPK/LKB1/mTOR signaling axes. Positioned upstream of mitochondrial injury, ferroptosis, and glucolipid dysmetabolism, the restoration of autophagy response may constitute a potential mechanism through which LT mitigates the aforementioned Cd-induced injuries (Figure 6). If these biological activities of LT are validated in human studies, LT holds promise as a cytoprotective adjuvant in clinical practice. Its value in combination with conventional Cd chelation therapy lies not in promoting Cd excretion but in repairing mitochondrial dysfunction and counteracting the ferroptotic cascade that chelators cannot reverse—primarily via the activation of the AMPK–autophagy axis—thereby accelerating hepatic functional recovery and potentially permitting dose-sparing of high-dose chelators to reduce their adverse effects. This mechanistically complementary strategy warrants further translational investigation. This promising therapeutic strategy and its potential clinical application warrant further investigation.

## Figures and Tables

**Table 1:** Biochemical parameters.

Biochemical Parameters	Groups			
	Ctrl	Cd	L-LT	H-LT
Serum TG (mmol/L)	1.2062 ± 0.3808	2.0439 ± 0.5785 **	1.5315 ± 0.4135	1.3190 ± 0.2586 #
Serum HDL-C (mmol/L)	5.0740 ± 0.8310	3.7716 ± 0.6370 **	4.2175 ± 1.4113	5.0140 ± 1.2456 #
Fasting blood glucose (mmol/L)	4.1793 ± 0.9463	5.7052 ± 1.2421 *	6.4257 ± 1.5873	6.4621 ± 1.2014
Liver TC (mmol/g prot)	0.2119 ± 0.0348	0.3175 ± 0.0520 **	0.2409 ± 0.0630 #	0.2790 ± 0.0561
Liver HDL-C (mmol/g prot)	0.0061 ± 0.0010	0.0049 ± 0.0008 *	0.0050 ± 0.0012	0.0064 ± 0.0012 #

Note: Data are presented as mean ± SEM (n = 9). * *p* < 0.05 and ** *p* < 0.01 compared to Ctrl group. # *p* < 0.05 compared to Cd group.

## Data Availability

The original contributions presented in this study are included in the article. Further inquiries can be directed to the corresponding author.

## Supplementary Materials

The following supporting information can be downloaded at: https://www.mdpi.com/article/10.3390/nu18183098/s1, Table S1: Composition of normal diet (Cat 1010088, Jiangsu Synergy Pharmaceutical Bioengineering Co., Ltd., Shanghai, China) (%). Table S2: Primers sequences used in the RT-qPCR.

## Abbreviations

The following abbreviations are used in this manuscript.

LT, *L*-theanine; Cd, cadmium; ROS, reactive oxygen species; mTOR, mammalian target of rapamycin; SIRT1, sirtuin 1; AMPK, 5′ AMP-activated protein kinase; LC3-II, light chain 3-II; Ctrl, control; FFA, free fatty acid; TG, triglyceride; TC, total cholesterol; HDL-C, high-density lipoprotein cholesterol; MDA, malondialdehyde; AKP, alkaline phosphatase; CAT, catalase; GSH, reduced glutathione; COX, cyclo-oxygen-ase; SDH, succinate dehydrogenase; DPP-4, dipeptidyl peptidase-4; *α*-SMA, alpha-smooth muscle actin; Col1*a*1, *alpha* 1 type I collagen; TGF *β*1, transforming growth factor *beta*1; Nrf2, nuclear factor erythroid 2-related factor 2; PGC-1*α*, peroxisome proliferator-activated receptor gamma coactivator 1*α*; INSR, insulin receptor; AKT, protein kinase B; p-AKT (Ser473), phosphorylation AKT (Ser473); PI3K, phosphoinositide-3-kinase; pPI3Kp85 (Tyr458)p55 (Tyr199), phosphorylation PI3K p85 (Tyr458)p55 (Tyr199); GPX4, glutathione peroxidase 4; FSP1, ferroptosis suppressor protein 1; ACC, Acetyl-CoA carboxylase; p-ACC (Ser79), phosphorylation ACC (Ser79); SREBP1, Sterol regulatory element-binding transcription factor 1; FASN, fatty acid synthase; CPT1*α*, carnitine palmitoyltransferase 1 *alpha*; PPAR*α*, peroxisome proliferator-activated receptor *alpha*; ACOX1, Peroxisomal acyl-Coenzyme A oxidase 1; GLUT2, glucose transporter 2; MT, metallothionein; ULK, Atg2/unc-51-like kinase complex; TXNIP, thioredoxin-interacting protein; NRF-1, nuclear respiratory factor 1; TFAM, mitochondrial transcription factor A.
