# Supplementary material for: L-Theanine Prevents Autophagy Impairment to Counteract Cadmium-Induced Hepatic Mitochondrial Dysfunction, Ferroptosis, and Glucolipid Dysmetabolism in Mice
Source: Nutrients. 2026 Sep 21;18(18):3098. doi: 10.3390/nu18183098 (PMC13610715; doi:10.3390/nu18183098)
Supplement: Supplementary file 1 [file nutrients-18-03098-s001.zip › nutrients-4488593-supplementary.pdf]

Supplementary materials for

L-Theanine preventing autophagy impairment to counteract cadmium-induced hepatic mitochondrial dysfunction, ferroptosis and glycolipid dysmetabolism in mice

Qiuyan Ban[1#], Wenjing Chi[#], Qiong Wang[1#], Junsheng Li[1], Yao Xia[1], Yue Meng[1], Mengru Li[1], Renliang Zhao[1], Yiding Yu[2*], Zhipeng Kan[3], Ning Li[4], Yan Ma[4], Xianqing Huang[4], Dongxu Wang[5*], and Guangshan Zhao[4*]

Table S1. Composition of normal diet (Cat 1010088, Jiangsu Synergy Pharmaceutical Bioengineering Co., Ltd., Shanghai, China) (%)

Ingredient	normal diet	
	g	kcal
Crude Protein	181.8	727.2
Crude Fat	47.1	424.2
Nitrogen-Free Extract	571.0	2283.8
Crude Fiber	⩽50.0	0
Crude Ash	⩽80.0	0
Water	⩽100.0	0
Total	1000	3530

g: gram, kcal: kilocalorie

Table S2. Primers sequences used in the RT-qPCR.

Gene	Forward (5'-3')	Reverse (5'-3')
MT1	ATGGACCCCAACTGCTCCT	AGCAGCAGCTCTTCTTGCAG
MT2	GCCTGCAAATGCAAACAATGC	CACTTGTCGGAAGCCTCTTTG
Nrf1	GCTTCGGGCATTTATCC	AACCCTTTGTTTTTGCTCTTC
Trx	CCTTCTTCCATTCCCTCTGTGAC	TTTCCTTGTTAGCACCGGAGAAC
TXNIP	AATACCCCTGACCTAATGGCACC	ATTCGAGCAGAGACTGACACACG
SOD1	AGATGACTTGGGCAAAGGTG	AATCCCAATCACTCCACAGG
LC3B	GAACCGCAGACGCATCTCT	TGATCACCGGGATCTTACTGG
CPT1α	GCAGACTGACATTGTGGACCTG	ATCTCCTGGCTGTCACCTTCTG
PPARα	CCTCAGGGTACCACTACGGAGT	GCCGAATAGTTCGCCGAA
ACOX1	ATTGGCACCTACGCCCAGAC	CCAGGCCACCACTTAATGGAA
SREBP1	CAAGGCCATCGACTACATCCG	CACCACTTCGGGTTTCATGC
PPARγ	CTCCAAGAATACCAAAGTGCGA	GCCTGATGCTTTATCCCCACA
α-SMA	CCCAGACATCAGGGAGTAATGG	TCTATCGGATACTTCAGCGTCA
Col1a1	GCTCCTCTTAGGGGCCACT	CCACGTCTCACCATTGGGG
COL3	CCTGGCTCAAATGGCTCAC	CAGGACTGCCGTTATTCCCG
CUGBP1	GTTCCAAGGACCTGGTCTGAG	GGACCTTCATGTTATGGAGAGCA
GAPDH	CTTTGGCATTGTGGAAGGGCTC	GCAGGGATGATGTTCTGGGCAG
